# Googling Allergy in Ireland: Content Analysis

**DOI:** 10.2196/16763

**Published:** 2020-05-13

**Authors:** Catherine King, Ciaran Judge, Aideen Byrne, Niall Conlon

**Affiliations:** 1 Department of Clinical and Laboratory Immunology St. James's Hospital Dublin Ireland; 2 Paediatric Allergy Department Our Lady's Children's Hospital, Crumlin Dublin Ireland

**Keywords:** allergy, food allergy, food intolerance, technology, Ireland, immunology

## Abstract

**Background:**

Internet search engines are increasingly being utilized as the first port of call for medical information by the public. The prevalence of allergies in developed countries has risen steadily over time. There exists significant variability in the quality of health-related information available on the web. Inaccurately diagnosed and mismanaged allergic disease has major downstream effects on patients, general practitioners, and regional allergy services.

**Objective:**

This study aimed to verify whether Ireland has a relatively high rate of web-based allergy-related searches, to establish the proportion of medically accurate web pages encountered by the public, and to compare current search results localized to Dublin, Ireland with urban centers elsewhere.

**Methods:**

Google Trends was used to evaluate regional interest of allergy-related search terms over a 10-year period using terms “allergy,” “allergy test,” “food allergy,” and “food intolerance.” These terms were then inputted into Google search, localizing them to cities in Ireland, the United Kingdom, and the United States. Output for each search was reviewed by two independent clinicians and deemed rational or nonevidence based, as per current best practice guidelines. Searches localized to Dublin were initially completed in 2015 and repeated in 2019 to assess for changes in the quality of search results over time.

**Results:**

Ireland has a persistently high demand for web-based information relating to allergy and ranks first worldwide for “allergy test,” second for “food allergy” and “food intolerance,” and seventh for “allergy” over the specified 10-year timeframe. Results for each of the four subsearches in Dublin (2015) showed that over 60% of websites promoted nonevidence-based diagnostics. A marginal improvement in scientifically robust information was seen in 2019, but results for “allergy test” and “food intolerance” continued to promote alternative testing 57% (8/14) of the time. This strongly contrasted with results localized to Southampton and Rochester, where academic and hospital-affiliated web pages predominantly featured. Government-funded Department of Health websites did not feature in the top five results for Dublin searches “allergy testing,” “food allergy,” or “food intolerance” in either 2015 or 2019.

**Conclusions:**

The Irish public demonstrates a keen interest in seeking allergy-related information on the web. The proportion of evidence-based websites encountered by the Irish public is considerably lower than that encountered by patients in other urban centers. Factors contributing to this are the lack of a specialist register for allergy in Ireland, inadequate funding for allergy centers currently in operation, and insufficient promotion by the health service of their web-based health database, which contains useful patient-oriented information on allergy. Increased funding of clinical allergology services will more meaningfully impact the health of patients if there is a parallel investment by the health service in information and communication technology consultancy to amplify their presence on the web.

## Introduction

### Background

The internet has become a major resource for people seeking information in relation to health. Overall, 57% of Irish adults search for health-related information on the web, and this most often applies to younger people and women [1].

The incidence of allergies has risen steadily in developed countries [2]. Food allergies are thought to affect approximately 1% to 3% of the Irish adult population and are a cause of significant public concern [3,4]. Allergic disease has long been a focus of public attention, with a nationwide questionnaire in 2015 demonstrating that 14% of Irish adults self-report allergic conditions (rhinitis, allergic eye disease, and food allergy, excluding allergic asthma) [5]. The diagnosis of allergic disease is centered upon a detailed clinical history and supported by the judicious use of tests to detect allergic sensitization [6]. Allergic sensitization is determined by skin prick testing or detection of allergen-specific immunoglobulin E (sIgE). When these test results conflict or there is a diagnostic doubt, gold standard investigation is advisable in the form of diagnostic challenges. These time-consuming procedures are only available in specialist centers.

The umbrella term of allergy is one that frequently attracts input from a variety of alternative medicine practitioners, naturopaths, homeopaths, and acupuncturists both in terms of diagnostic testing and claims to treatment. Despite the existence of scientifically robust and evidence-based allergy tests, alternative approaches to diagnosis are widely used by the public, with attempts at regulatory control being previously described as “woefully inadequate” [2]. Alternative tests, including food-specific immunoglobulin G testing, kinesiology, hair analysis, Vega testing, and leukocytotoxic tests, are widely available, lack scientific basis and diagnostic rigor, and have been discredited by a variety of governmental, professional, and expert bodies internationally [6-9]. The use of complementary and alternative medicine (CAM) as a treatment modality for allergy has also been increasingly reported, with 37% of people with allergic disease using CAM during the preceding 12 months in a recent European study [10]. In 2018, 60% of surveyed allergists in the United States had patients who encountered adverse reactions from the use of CAM, with 81% of respondents encountering patients who discontinued conventional therapy while using CAM, irrespective of medical advice [11]. Despite this, alternative approaches continue to be advocated by some health care professionals, including registered medical practitioners. The risks of a misdiagnosis of food allergy include inappropriate dietary restrictions and negative quality-of-life consequences, the misattribution of symptoms to allergic diseases resulting in delayed assessments, the inappropriate fear of life-threatening reactions, and direct and indirect financial costs related to these risks [12].

### Objectives

The volume of easily accessible information available on the web offers an excellent opportunity to provide helpful, evidence-based information and services to the information-seeking public. In this study, we sought to examine the web-based information sources accessible to members of the Irish public who were seeking information on allergy testing. We first interrogated allergy testing search requests by Irish internet users. We then examined the prominent sites presented when searching for allergy tests in an Irish setting and determined whether they promoted rational or alternative testing approaches. We initially reviewed these search requests in 2015 and repeated the study using identical search terms in 2019 to identify whether the standard of information available to internet users had changed over time. Finally, we compared the 2019 Irish results with similar UK and US populations using identical search terms.

## Methods

Google Trends was used to evaluate interest by region of allergy-related search terms over a 10-year period (January 1, 2009, to December 31, 2018) [13]. The search terms used were “allergy” and related subsearches “allergy test,” “food allergy,” and “food intolerance.” The period of trend analysis was purposely predated to the start of 2019 so that our subsequent searches in July 2019 did not interfere with the trend results. Google Trends data provide a list of countries ranked by the relative popularity of the specified search term, as a proportion of total searches in each country.

Each of the search terms was inputted into Google search and the output reviewed. Output webpages were reviewed independently by two clinicians and classified as *rational*, if the services or information offered were based on clinical history and standard sensitization testing, or *alternative,* if the practitioners offered any non–evidence-based approaches, as outlined in the Irish Food Allergy Network and Irish Association of Allergy and Immunology position statement [7]. Results localized to country level (Ireland) were reviewed initially. Then, a comparison of local results from Dublin, Southampton (United Kingdom), and Rochester (United States) was performed. Sponsored advertisements, forums/discussion groups, duplications, and reports on news or weather were excluded from analysis. Analysis was limited to Google search pages one and two.

## Results

### Trend Analysis of Allergy-Related Searches Worldwide

Data from Google trends indicate that Ireland has a persistently high demand for information regarding allergy. Trends from 2008 to 2019 demonstrate that Ireland ranks seventh in the world in searches for the term allergy (Table 1). Analysis of data for the subsearch term “allergy test” shows that Ireland is ranked first in the world for this particular search over the timeframe, and second in the world for both other subsearch terms “food allergy” and “food intolerance” [13]. These results demonstrate a high demand for web-based information regarding allergies from the Irish population.

**Table 1:** Google Trends January 1, 2009, to December 31, 2018, worldwide for the specified search terms.

Search term	Worldwide ranking			Ireland ranking
	First place	Second place	Third place	
“Allergy”	United States	Canada	Philippines	7
“Allergy test”	Ireland	Singapore	United States	1
“Food allergy”	United States	Ireland	Australia	2
“Food intolerance”	Malta	Ireland	Australia	2

Analysis of localized Google search engine results for Ireland (search term “allergy Ireland”) in 2015 revealed that alternative diagnostic services and information featured highly (Table 2). At that time, 63% (10/16) of included results were from private companies selling non–evidence-based commercial tests, 19% (3/16) were from private medical services with rational testing procedures, and the remaining 19% (3/16) were from organizations offering evidence-based patient information. Regional localization to Dublin and analysis of the subsearch terms also confirmed the prominence of websites endorsing alternative approaches to allergy diagnostics. Furthermore, 69% (11/16) websites listed under an “allergy test Dublin” Google search promoted alternative non–evidence-based approaches to allergy. Use of the search term “food allergy Dublin” provided similar results, with 67% (10/15) of included results relating to alternative health care websites. The disparity was greater again when “food intolerance Dublin” was used as a search term, with just 13% (2/15) of included webpages promoting a rational assessment approach versus 87% (13/15) promoting alternative approaches.

**Table 2:** Summary of results of localized internet searches for the specified allergy-related search terms.

Search term, by region		Website results, n (%)		
		Non–evidence-based practices	Medical facility, rational testing	Evidence-based guidelines, journals
“**Allergy”**				
	Ireland (2015)	10 (63)	3 (19)	3 (19)
	Ireland (2019)	1 (9)	5 (46)	5 (46)
	Southampton, United Kingdom (2019)	1 (11)	4 (44)	4 (44)
	Rochester, United States (2019)	0 (0)	7 (100)	0 (0)
“**Allergy Test”**				
	Dublin (2015)	11 (69)	5 (31)^a^	N/A^b^
	Dublin (2019)	8 (57)	5 (36)	1 (7)
	Southampton, United Kingdom (2019)	1 (8)	8 (67)	3 (25)
	Rochester, United States (2019)	0 (0)	7 (70)	3 (30)
“**Food Allergy”**				
	Dublin (2015)	10 (67)	5 (33)^a^	N/A
	Dublin (2019)	6 (46)	5 (39)	2 (15)
	Southampton, United Kingdom (2019)	1 (9)	4 (36)	6 (55)
	Rochester, United States (2019)	0 (0)	5 (50)	5 (50)
“**Food Intolerance”**				
	Dublin (2015)	13 (87)	2 (13)^a^	N/A
	Dublin (2019)	8 (57)	4 (29)	2 (14)
	Southampton, United Kingdom (2019)	3 (27)	6 (55)	2 (18)
	Rochester, United States (2019)	1 (11)	3 (33)	5 (56)

^a^The 2015 search results for “Allergy test Dublin,” “Food allergy Dublin,” and “Food intolerance Dublin” were recorded as either alternative or evidence based only.

^b^N/A: not available.

### Subsearch Terms Localized to Ireland (2019)

A greater proportion of evidence-based information was noted upon repeating these Google searches in July 2019. Current searches for “allergy Ireland” result in 9% (1/11) from a private company selling non–evidence-based commercial tests, 45% (5/11) from private health care facilities engaged in rational testing, and a further 45% (5/11) from organizations offering evidence-based patient information. However, searches for “Allergy test Dublin” continued to promote alternative approaches to diagnosing allergy, with 57% (8/14) of the results coming from private companies selling non–evidence-based commercial tests. Results for “Food allergy Dublin” also continued to feature a high proportion of alternative testing approaches (6/13, 46%). This also was seen in searches for “Food intolerance Dublin,” which had 57% (8/14) results endorsing non–evidence-based diagnostics.

### Regional Differences in the Proportion of Nonevidence-Based Webpages Encountered

Comparison of the 2015 and 2019 results showed a marginal improvement in the availability of rational, scientific information regarding allergy over this timeframe. However, when the Irish search results from 2019 were evaluated against current search results for comparable UK and US populations, significant inadequacies are highlighted. Data were generated with identical search terms localizing to Southampton (United Kingdom) and Rochester (United States). Assessment of these results indicated that the vast majority of websites generated for each search pertained to websites promoting rational evidence-based approaches to assessment. A preponderance of academic and public hospital webpages was noted. Searches for “allergy Rochester New York,” “allergy test Rochester New York,” and “food allergy Rochester New York” resulted solely in rational testing approaches and scientifically robust information, without any non–evidence-based websites featuring.

## Discussion

### The Increasing Burden of Allergic Disease

The incidence of allergic conditions in both developed and developing countries has been increasing for over 50 years. One-third of people in the United Kingdom are estimated to suffer symptoms related to allergy at some stage during their lives [2]. The use of the internet has also risen steadily over recent generations, especially involving the investigation of medical conditions on the web. Over 70% of internet users in the United States have stated that they look on the web for health information, with over three-quarters of these queries beginning on a web-based search engine such as Google [14].

The increase in demand for web-based information creates both challenges and opportunities. Appropriate use of the internet enables health services to positively affect the lives of many people living with allergies. People who access health information on the internet have been shown to be more likely affected in their offline approach to health care [15], which in turn impacts on the provision of health care services. Figures on the extent of costs are difficult to obtain in Ireland; however, allergic disease accounts for 6% of general practitioner (GP) consultations in the National Health Service (NHS), 0.6% of hospital admissions, and 10% of GP prescribing budget. The cost (excluding hospital services) to the NHS is approximately £900 million (US $1.2 billion) per year [16]. Inadequately treated allergic disease also leads to a significant reduction in economic productivity with €55 to €151 billion (US $60 to $164 billion) lost per annum in the European Union, according to a 2014 study [17].

### Complementary and Alternative Medicine Practitioners

When members of the Irish public search for guidance on the web regarding allergic disease, they are faced with a large volume of alternative testing approaches, often taking precedence over results for rational services. This is rightly a cause for concern. Previous studies in North America show that 91.5% of users will select a website from the first page and that the likelihood of a user clicking a result on the third page is 1.1% [18]. The ease of access to non–evidence-based information has the potential to promote costly practices and to increase the pressure on an already burdened health system through mismanagement of allergic diseases. There are countless medical conditions that attract input from non–evidence-based sources, but allergology has consistently shown itself to be an area of particular interest among CAM practitioners. A study of the websites of over 300 alternative health care providers demonstrated that 85% of naturopaths offer diagnosis, treatment, or efficacy for specifically treating allergic disease or sensitivity [19]. Similarly, a 2018 study investigating the educational quality of 300 food allergy YouTube videos showed that alternative medicine providers were the most common source of such content, with almost half of the videos depicting non–IgE-mediated reactions and frequently recommending controversial diagnostics [20]. In contrast to the word “allergy,” which is frequently misappropriated by such websites, a recent study of worldwide internet search results for “anaphylaxis” showed that links to well-established, evidenced-based information were far more often seen [21].

### Unmet Clinical Need in Allergic Disease

The internet has created a means of positively impacting the lives of many individuals affected by allergies while simultaneously decreasing the costs placed on the Irish health service. However, these potential benefits rely on the availability of accurate, evidence-based, and accessible resources that are provided by experts in allergology. There is a wide variation in the amount of allergologists working in different European countries, with a mean of 1.81 specialists per 100,000 inhabitants [22]. Figures for Ireland are not directly comparable as the country does not have a recognized training scheme for allergy. With a rate of 0.14 immunologists (pediatric and adult) per 100,000 citizens, who also have commitments to laboratory work, immunodeficiency, and autoimmunity, the relative time dedicated solely to allergy is assumed to be far below the European average [23]. Given that allergy is not currently a recognized medical specialty in Ireland, it should come as no surprise that tertiary allergy referral centers make up a relatively small proportion of localized web-based searches and that services run by GPs are often seen. Much has been previously published on how best to alleviate the pressure on chronically underresourced tertiary allergy departments [24]. A common theme throughout these (predominantly UK-based) reports is improved support and education of primary care physicians in the field of allergy. Proposed interventions have included development of a network of GPs with special interest in allergy [25,26], a core allergy curriculum for all GPs [27], and better training of nurses, pharmacists, and dieticians to enable them to advise patients in the community [28].

### Future Directions in Allergic Disease Management: Investment in Information Technology Resources

Although these proposals are commendable and steps should certainly be taken in their establishment, there can be no doubt that these are long-term investments and change will be slow to occur. There has also been growing interest in the use of information technology (IT)–based interventions, such as telemedicine assessments in adult allergy [29], app-based monitoring of allergic rhinitis [30], and a pilot program of email communication between allergists and nonspecialists for new referrals to allergy clinics [31], with further prospective studies required. Innovative and accessible approaches to delivering allergy services are required in the setting of an inadequately resourced system, where the lack of timely care for patients has undoubtedly contributed to people seeking out alternative practitioners. There is an undeniable need for increased support and funding for dedicated allergy services currently in operation in Ireland. However, to create a meaningful impact on patients’ health-seeking behaviors, we need a parallel investment in IT services currently in use by the Irish health service.

In 2019, the information and communication technology capital allocation for the Irish health service was €85 million (US $92 million), making up approximately 0.5% of the total health care budget of over €16 billion (US $17 billion) [32]. The Health Service Executive (HSE) website was given a radical overhaul in 2013, which included development of a web-based database of over 600 health conditions and treatments. This database (entitled *Health A-Z*) contains useful information on the diagnosis and management of allergy and highlights the existence of alternative testing approaches, unambiguously describing them as unproven, unreliable, and best avoided by the public [33]. The content for this sizeable information resource was provided to the HSE completely free of charge from the NHS Choices website in the United Kingdom. Details on the volume of internet traffic to the HSE *Health A-Z* are not readily accessible to the public on the web. In our subsearch results for Dublin in 2019 (for “allergy test,” “food allergy,” and “food intolerance”), the HSE website never featured within the top 5 results. The question of cost efficacy and suboptimal internet traffic is certainly an issue that has been raised regarding the prototypic NHS Choices website in the past. A report published several years after its launch evaluated the NHS Choices website against the average website of a US company at the time (with a similar volume of monthly visitors) and found that the UK Department of Health was spending nearly four times more on site management, hosting costs, and development than their counterparts [34]. The downstream effect of this was the NHS entering a partnership with a digital marketing agency in 2012, which was specializing in analysis of consumer behaviors. This led to a significant improvement in their volume of web-based traffic [35]. The Irish health system, having saved huge costs in the development of their web-based health database, would undoubtedly benefit from external IT consultancy to improve their reach and strengthen their web-based presence. A public that is well informed and empowered in matters of their own health is a worthy long-term investment, and this applies to all areas of medicine, not solely allergology.

### Conclusions

This study provides a snapshot of the information obtained when searching for information relating to allergy on the internet. There is great potential to provide accurate and evidence-based guidance to an increasing population, which would maximize the appropriate use of allergy services in Ireland. Unfortunately, this is not currently the case. Results for costly, non–evidence-based services predominate when searching on the web in Ireland, which differs relative to the United Kingdom and United States. There is a great need to improve the provision of allergy services in Ireland and to educate the Irish public on allergic disease, and investment in local internet resources is central to this endeavor.

## Abbreviations

CAM: complementary and alternative medicine
GP: general practitioner
HSE: Health Service Executive
IT: information technology
NHS: National Health Service
